# Reconstructing Eocene Antarctic river drainage from provenance analysis of Amundsen Sea embayment sediments

**DOI:** 10.1126/sciadv.aea2373

**Published:** 2025-12-10

**Authors:** James W. Marschalek, Tina van de Flierdt, Christine S. Siddoway, Stuart N. Thomson, Guy J. G. Paxman, Stewart S. R. Jamieson, Ethan Conrad, Kathy J. Licht, Sidney R. Hemming, Michael J. Bentley, Claus-Dieter Hillenbrand, James A. Smith, Johann P. Klages, Matthew Fox, Guido Pastore, Pieter Vermeesch

**Affiliations:** ^1^ Department of Earth Science and Engineering, Imperial College London, Exhibition Road, London, SW7 2BP, UK.; ^2^ Department of Geology, Colorado College, Colorado Springs, CO 80903, USA.; ^3^ Department of Geosciences, University of Arizona, 1040 E. 4th Street, Tucson, AZ 85721, USA.; ^4^ Department of Geography, Durham University, South Road, Durham DH1 3LE, UK.; ^5^ London Geochronology Centre, Department of Earth Sciences, University College London, Gower Street, London WC1E 6BT, UK.; ^6^ Department of Earth and Environmental Sciences, Indiana University Indianapolis, Indianapolis, IN 46202, USA.; ^7^ Lamont-Doherty Earth Observatory, Columbia University, Palisades, NY 10027, USA.; ^8^ British Antarctic Survey, High Cross, Madingley Road, Cambridge CB3 0ET, UK.; ^9^ Department of Geosciences, Alfred-Wegener-Institut Helmholtz-Zentrum für Polar- und Meeresforschung, Bremerhaven, Germany.; ^10^ Laboratory for Provenance Studies, Department of Earth and Environmental Sciences, University of Milano-Bicocca, Piazza della Scienza, 20126 Milano, Italy.

## Abstract

Sedimentary records can illuminate relationships between the climate, topography, and glaciation of West Antarctica by revealing its Cenozoic topographic and paleoenvironmental history. Eocene fluvial drainage patterns have previously been inferred using geochemical provenance data from an ~44– to 34–million year deltaic sandstone recovered from the Amundsen Sea Embayment. One interpretation holds that a low-relief, low-lying West Antarctic landscape supported a >1500-kilometer transcontinental river system. Alternatively, higher-relief topography in central West Antarctica formed a drainage divide between the Ross and Amundsen seas. Here, zircon U-Pb data from Amundsen Sea Embayment sediments are examined alongside known regional bedrock provenance signatures. These analyses suggest that all observed provenance indicators in the Eocene sandstone derive from West Antarctic rocks. This implies that a local river system flowed off a West Antarctic drainage divide, helping constrain the mid-Late Eocene evolution of West Antarctic topography with implications for the history of rifting and the characteristics of sediments infilling interior basins.

## Teaser

Amundsen Sea detrital zircon U-Pb data support a high-relief, drainage-dissected West Antarctic topography in the Eocene.

## INTRODUCTION

The topography of West Antarctica has shaped the history of its glaciation (*1*–*3*). From the end of the Eocene to the beginning of the Miocene, paleotopographic reconstructions suggest that large portions of West Antarctica were above sea level, enabling an ice sheet to grow in warmer conditions than would be possible today (*4*–*6*). To understand how the West Antarctic Ice Sheet (WAIS) developed and, once established, responded to changes in environmental conditions, it is important to consider the timing and nature of this transition from a subaerial topography to the modern subglacial basins down to ~2500 m below sea level. Reconstructions of West Antarctic topography from before the formation of the WAIS are thus key when seeking to understand the early history of this ice sheet.

However, in the Eocene, there is substantial uncertainty regarding the topography of West Antarctica. Reconstructions for the Eocene-Oligocene boundary (*4*, *6*) use restoration of offshore sediment volumes observed in seismic data (*7*) to reconstruct the landmass above sea level. This results in an unrealistically smooth model surface that is inconsistent with isolated observational data. Such observations include the occurrence of Eocene marine diatom species beneath a modern West Antarctic ice stream, implying that the site was already below sea level in the Eocene (*8*), and a pre-Late Oligocene paleosol drilled at Deep Sea Drilling Project Site 270, indicating subaerial exposure of a Ross Sea basement high (*9*). Debris from the basal ice of the Byrd ice core in central West Antarctica also suggest a pre-Oligocene terrestrial environment locally (*10*).

These examples point to more complex preglacial regional topography than is currently captured in continental-scale reconstructions for the Eocene-Oligocene boundary (*4*, *6*), with broad basement highs and intervening rift basins produced by extensional tectonism that began in the Cretaceous (*5*). Such features persist beneath the Ross Ice Shelf (*11*) and in central West Antarctica (*12*). Basement highs represent (subsidence-restored) regions that were likely higher than those suggested by Eocene-Oligocene boundary reconstructions and, thus, sites of erosion. In contrast, rift basins were likely lower than in the reconstructions, forming submarine areas that were depocenters. Direct geological constraints offer the means to unravel such complexities, but these remain very rare because of sediment removal by later glacial erosion, burial by large thicknesses of sediment, or cover by the present-day ice sheet.

**Fig. 1.**
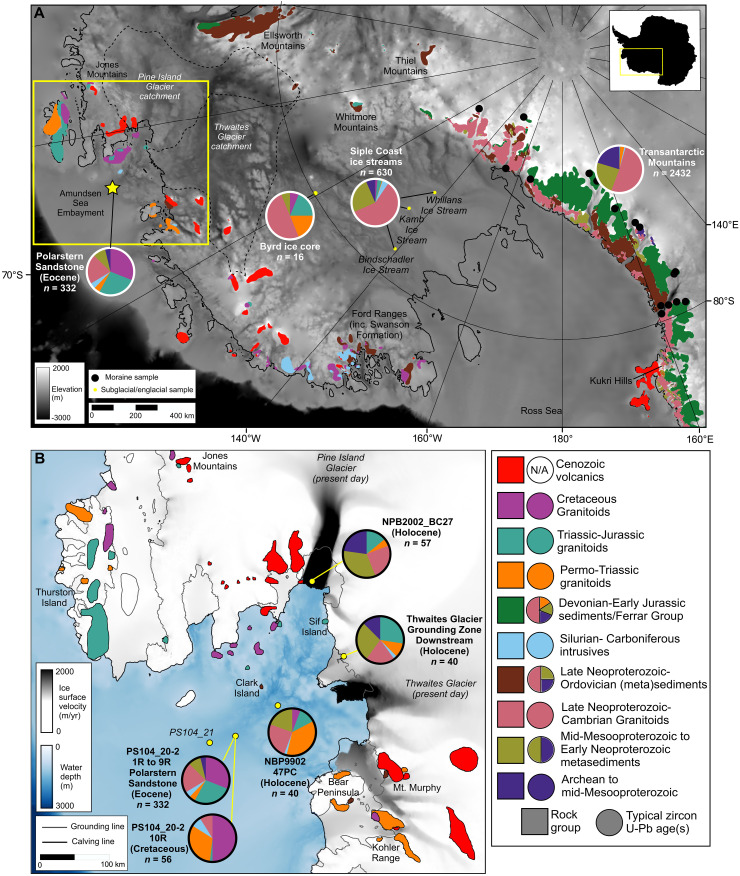
Map of main geological source areas in West Antarctica. (**A**) Geology is colored according to the major rock groups present (*95*). Qualitative pie charts in the legend indicate the typical zircon U-Pb ages shed from each rock group, showing repeated recycling of older zircons in sedimentary rocks. Pie charts (A) represent unconsolidated sediment zircon U-Pb ages from the following: Transantarctic Mountains moraines (*n* = 2432) (*32*–*36*, *65*), Siple Coast ice stream subglacial tills (*n* = 630) (*34*), Byrd ice core basal debris (*n* = 16) (*10*), and the Middle-Late Eocene Polarstern Sandstone (*n* = 332) (*14*). Pie chart bins encompass the main age populations in rock groups (see key) and are >1300, 1300 to 650, 650 to 450, 450 to 300, 300 to 230, 230 to 150, and <150 Ma. The modern ice-sheet grounding line is plotted in black, and the Pine Island and Thwaites Glacier catchments are shown by dashed lines (*96*, *97*). The base map for both panels shows modern BedMachine bed topography (*98*). (**B**) The location of zircon U-Pb samples in the Amundsen Sea Embayment, with pie charts and geology colored as in (A). Ice-surface velocities show the location of modern glaciers (*99*). The yellow box on (A) indicates the extent of this panel. Grounding line (gray lines) and calving front (black lines) positions are from the Scientific Committee on Antarctic Research Antarctic Digital Database, accessed in 2022 (*100*). Note that zircons in Holocene sediments may be recycled from older sediments. yr, year.

Nevertheless, one such geological constraint was obtained during the 2017 RV *Polarstern* Expedition PS104 to the Amundsen Sea using the Seafloor Drill Rig MARUM-MeBo70 (*13*). Drill site PS104_20-2 in the inner Amundsen Sea Embayment (Fig. 1) recovered core material from a mid-Late Eocene [44 to 34 million years ago (Ma)] river-transported coastal deltaic sandstone, named the “Polarstern Sandstone” (*14*). The Polarstern Sandstone rests unconformably upon underlying mid-Cretaceous (92 to 83 Ma) terrestrial sediments, which are capped by a thin layer of indurated lignite (*15*). Study of the Polarstern Sandstone offers a valuable opportunity to constrain Eocene West Antarctic environmental conditions and topography. By comparing provenance data from the Polarstern Sandstone (i.e., its geochemical signature) to equivalent data from rock exposures and unconsolidated sediments around West Antarctica, the spatial extent of the river catchment can be reconstructed.

If the Polarstern Sandstone contains detritus derived purely from proximal bedrock sources in West Antarctica, this would be in accord with the broad pattern of preglacial Eocene-Oligocene boundary topographic reconstructions. These show an elevated region in the center of West Antarctica forming a drainage divide between the Amundsen and Ross seas (hereafter the “high-relief model”) (*4*, *6*). Conversely, provenance data indicating detrital input from distant source rocks, such as in the Transantarctic Mountains, would imply that a low-lying, low-relief landscape succeeded the rift topography, supporting a >1500-km transcontinental river system (hereafter the “low-relief model”) (*14*). Identifying which of these two potential topographic configurations is more likely will have substantial consequences for our understanding of the long-term evolution of West Antarctic topography, including the elevation and subsidence history of this continental-scale rift province (*16*), influence of elevation on initiating glaciation at the onset of Oligocene “icehouse” conditions (*1*, *3*), and the characteristics of sediment infilling of interior basins (*17*).

To test the two hypotheses, we present previously unidentified constraints on the spatial extent of West Antarctic rivers that drained into the Amundsen Sea during the Eocene. These constraints are derived from comparison of the provenance characteristics of the Polarstern Sandstone—including zircon, apatite and rutile U-Pb dates, zircon Hf isotope data, and apatite fission track (AFT) dates (*14*)—to new zircon U-Pb data from older mid-Cretaceous terrestrial sediments from the same site, and Holocene marine and sub-ice shelf sediments from close to the core site in the Amundsen Sea Embayment (see Materials and Methods). We augment these analyses with a compilation of published geochronological datasets from West Antarctica and the Transantarctic Mountains, including unconsolidated sediments and selected bedrock sources. This comparison to source areas helps test whether the sediments deposited as the Polarstern Sandstone are consistent with smaller regional river catchments confined to West Antarctica (high-relief model), or whether they indicate Eocene transcontinental sediment transport through an integrated drainage system flowing across the West Antarctic rift province (low-relief model).

## RESULTS

### Zircon U-Pb data

**Fig. 2.**
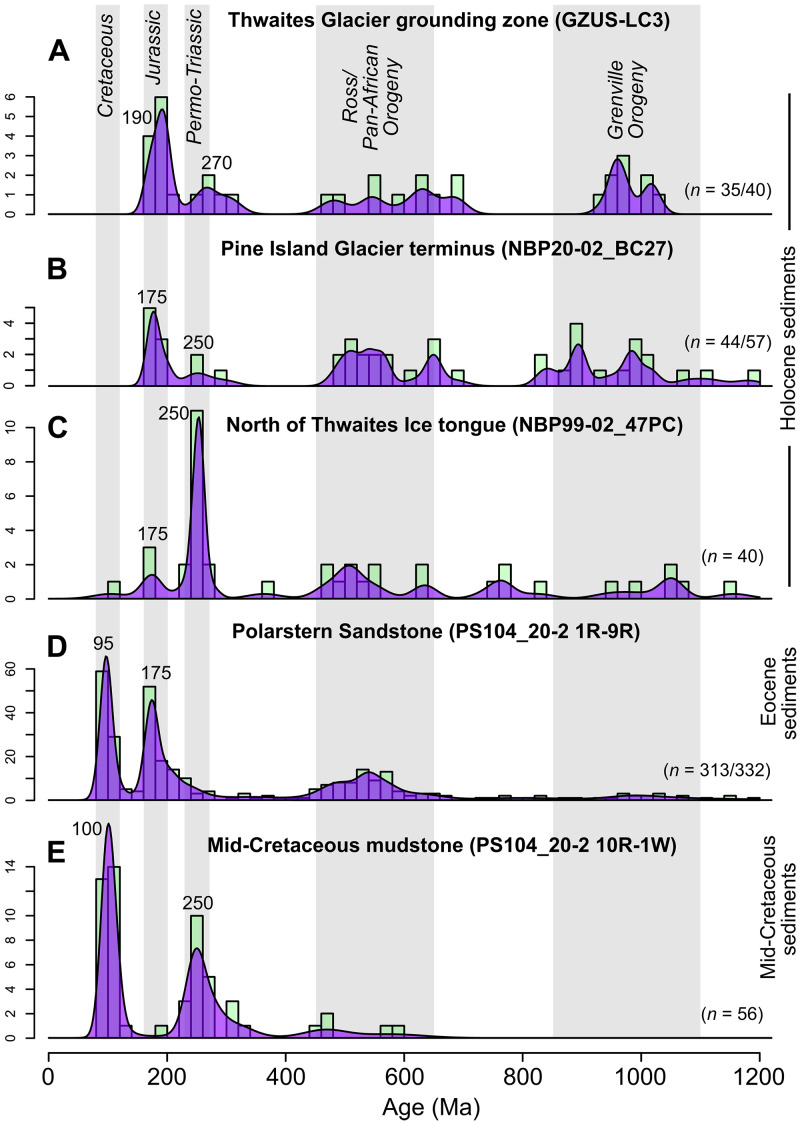
Kernel density estimates of new detrital zircon U-Pb ages from the Amundsen Sea Embayment. Plotted using IsoplotR (*92*) with a bandwidth of 15 Myr, overlain on histograms with a 20-Myr bin. Data are from (**A**) Thwaites Glacier grounding zone (GZUS-LC3), (**B**) Pine Island Glacier terminus (NBP20-02_BC27), (**C**) north of Thwaites Glacier Tongue (NBP99-02_47PC), (**D**) the Polarstern Sandstone (PS104_20-2 sections 1R to 9R) (*14*), and (**E**) mid-Cretaceous mudstone (PS104_20-2 10R-1 W). The Polarstern Sandstone data (*14*) were reprocessed to ensure consistency with our new data (see Materials and Methods) and are combined from seven samples at different depths in the core. Dates older than 1200 Ma are not plotted. The number of grains displayed in the plots (i.e., <1200 Ma) of the total number of concordant analysis is indicated in the bottom right of each plot. The age of Mid-Late Phanerozoic peaks was identified visually from kernel density estimates and rounded to the nearest 5 Myr. Note that for the Polarstern Sandstone, our ~175-Ma estimate of the modal age differs from the previously published ~190-Ma age peak estimate (see Materials and Methods) (*14*).

Holocene sediments in the Amundsen Sea Embayment can be analyzed to assess the U-Pb ages of zircons currently being discharged into the embayment (Fig. 2). Three Holocene sediment samples were analyzed from core NBP99-02_47PC (74.21°S, 106.28°W) north of Thwaites Glacier, core NBP20-02_BC27 (75.02°S, 100.75°W) close to the terminus of Pine Island Glacier, and core GZUS-LC3 near the Thwaites Glacier grounding zone (75.21°S, 104.83°W) (Fig. 1B). The latter two samples were collected as part of the Thwaites Offshore Research project of the International Thwaites Glacier Collaboration.

The sample from the Pine Island Glacier terminus (core NBP20-02_BC27, *n* = 57) contains two distinct zircon U-Pb age populations spanning ~200 to 165 and ~300 to 240 Ma (Fig. 2B). The sample also contains notable populations at ~650 to 450 and ~1100 to 800 Ma, plus 13 grains dating to >1200 Ma.

The Thwaites Glacier grounding zone sample contained a broadly similar range of age populations compared to the Pine Island Glacier sample (Fig. 2A, *n* = 40). The largest population (*n* = 11) spans ~210 to 165 Ma. An age population spanning ~310 to 250 Ma (*n* = 5) is also present. Similar to the Pine Island Glacier sample, there are ~650 to 450 and ~1100 to 850 Ma ages present (*n* = 8 and 9 grains, respectively), with five grains dating to >1200 Ma.

Further from the coast, the sample from core NBP99-02_47PC is dominated by a large population of Permo-Triassic ages, spanning ~275 to 225 Ma (*n* = 15; Fig. 2C). A younger age population was identified at ~180 to 170 Ma (*n* = 5), and older populations at ~650 to 450 Ma (*n* = 10) and ~1100 to 950 Ma (*n* = 5). A single Cretaceous (~104 Ma) grain was analyzed, and no concordant grains were older than 1200 Ma.

The Holocene sediments analyzed provide insight into subglacial rock sources close to the Polarstern Sandstone core site (PS104_20-2) at the present day, which likely formed subaerial coastal exposures during the Eocene. Holocene sediments could, however, contain recycled zircons eroded from the Polarstern Sandstone by Cenozoic glacial and fluvial processes, as seismic reflection data from the Amundsen Sea Embayment indicate a considerable thickness and lateral extent of seismographic units of Eocene age (*15*). To address this problem, we collected zircon U-Pb data from mid-Cretaceous (92 to 83 Ma) sediments from Site PS104_20 (*15*). The sample comprises terrestrial mudstone taken from core 10R-1 W (111 to 126 cm), which lies stratigraphically below the Polarstern Sandstone and predates the sandstone by at least 40 million years (Myr). The zircon U-Pb data from the 10R-1 W interval are dominated by an age peak at ~100 Ma (~50% of grains), with a second broader age population peaking at ~250 Ma (Fig. 2E). Four dated grains span ~330 to 305 Ma, but the number of analyses is too low to confidently say whether this reflects a distinct age population or a tail of the population peaking at ~250 Ma. Five concordant grains were dated to 600 to 450 Ma, with no older zircons analyzed.

### Hydrological modeling

**Fig. 3.**
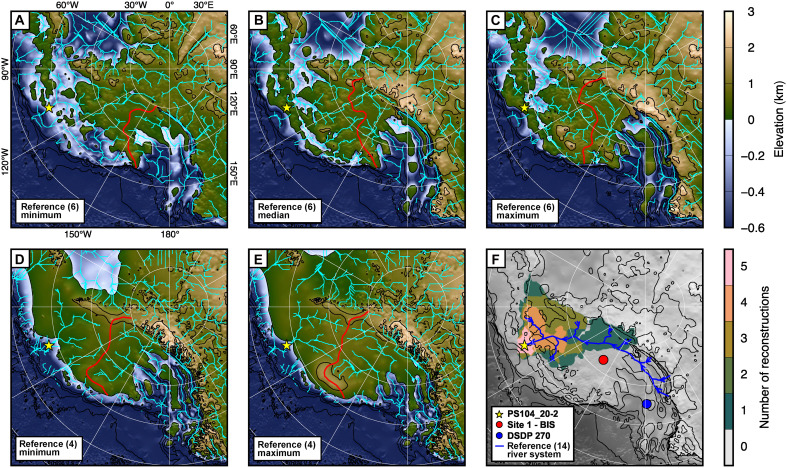
Hydrological modeling on reconstructed 34-Ma topographies. Plotted are the minimum (**A**), median (**B**), and maximum (**C**) reconstructions from reference (*6*), and minimum (**D**) and maximum (**E**) reconstructions from reference (*4*). These are superimposed with pale blue lines marking modeled hydrological flow pathways for each topography, assuming water follows the path of steepest descent. All topographies are referenced to present-day sea level and the contour interval is 1 km. The red line indicates the drainage divide between Ross Sea and Amundsen Sea catchments. Site PS104_20-2 is shown as a yellow star and has been rotated to correct for post–34 Ma plate motion (*78*). Before the hydrological routing calculation, internal “sinks” within the topographies were filled to remove enclosed topographic lows that would otherwise cause discontinuities in the calculated flow network. (**F**) Heatmap of the extent of the modeled upstream drainage catchment of the PS104_20-2 core site across the five topographic reconstructions. To illustrate spatial relationships, this is overlain on a grayscale version of (B) topography. Site 1 (Bindschadler Ice Stream, BIS) and Deep-Sea Drilling Project Site 270 are shown as a red dot and blue dot, respectively (*8*, *9*). Both sites have also been rotated to correct for post–34 Ma plate motion (*78*). The river system proposed to flow from the Transantarctic Mountains to the Amundsen Sea over the low relief model topography is shown by the blue line (*14*).

The new zircon U-Pb data from the Amundsen Sea Embayment help identify sediment source regions for the Polarstern Sandstone. To help convert these interpretations into inferences regarding drainage networks and paleotopography, we simulate hydrological drainage pathways on five preglacial (Eocene-Oligocene boundary, ~34 Ma) topographies (Materials and Methods; Fig. 3) (*4*, *6*). These represent high-relief model scenarios. We opt to plot topography relative to modern sea level to avoid uncertainties associated with calculating local Antarctic relative sea-level change, which will deviate notably from the global average due to glacial isostatic adjustment in an ice sheet–free world (*18*). Global mean sea level in the Middle-to-Late Eocene was up to 80 m higher than present (*19*), but this difference is insufficient to markedly change coastlines at the regional scale of this study.

All the simulated hydrological catchments are confined to West Antarctica (Fig. 3). The Polarstern Sandstone core site is typically close to the coast, in general agreement with the coastal deltaic depositional setting. Specific river catchments pass over the Thurston Island Crustal Block (exposed at Thuston Island, the Jones Mountains, and the eastern coast of Pine Island Bay; Fig. 1) and flow from the West Antarctic interior beneath the modern Thwaites Glacier, routing sediment toward the Polarstern Sandstone core site (Fig. 3). Catchments draining toward the Amundsen Sea are smallest for the median and maximum reconstructions of (*6*) because of the low topography in the region currently overlain by Pine Island Glacier (Fig. 3, A and B).

## DISCUSSION

### Zircon U-Pb signature of Holocene Amundsen Sea Embayment sediments

The new zircon U-Pb data allow comparison between the ages of bedrock sources contributing detritus to the Eocene fluvio-deltaic Polarstern Sandstone and the modern deposits in the inner Amundsen Sea Embayment (dominated by detritus from Pine Island Glacier and Thwaites Glacier). The detrital zircon U-Pb age spectra for the Holocene sediments reveal four main zircon U-Pb age populations (Fig. 2). Two Phanerozoic populations are ~190 to 170 and ~275 to 230 Ma, with Neoproterozoic–Early Paleozoic populations spanning ~650 to 450 and ~1100 to 850 Ma.

Only one concordant Cretaceous (~120 to 90 Ma) zircon was dated in our Holocene samples, with none in our Thwaites Glacier sample (Fig. 2). Although this Thwaites Glacier sample was limited to only 40 concordant analyses, this gives 95% confidence that a Cretaceous population, if present, comprises less than 7% of the zircons (*20*). This observation contrasts with surface sediments to the north of Thwaites Glacier, which contains biotite grains with a modal ^40^Ar/^39^Ar age of ~115 Ma (*21*). This unexpected result may indicate that the ~115-Ma old biotite grains originate from bedrock beneath smaller glaciers to the east or west (Figs. 1B and 2B) and were rafted to the north of Thwaites Glacier by icebergs entrained in the coastal current. Pine Island Glacier is unlikely to be the source of the ~115-Ma old biotite grains as no grains of this age were detected near its terminus (*n* = 30) (*21*). Alternatively, rocks bearing ~115 Ma biotite grains beneath Thwaites Glacier could have a low zircon fertility, or the ~115-Ma ^40^Ar/^39^Ar ages could reflect thermal resetting of the biotite K-Ar decay system (*22*) during magmatism at ~100 Ma (*23*). Applying both zircon U-Pb and biotite ^40^Ar/^39^Ar dating to individual bedrock samples could test this hypothesis. The lack of Cretaceous zircons in the Holocene samples contrasts with the abundance of these ages in the mid-Cretaceous and Eocene samples (Fig. 2) (*14*).

A Jurassic (~190 to 160 Ma) zircon U-Pb population is measured in Holocene sediments from all three sites in the inner Amundsen Sea Embayment (Fig. 2). This age group typifies rocks outcropping locally on Thurston Island (*24*), around Pine Island Bay (*25*), and the granitic bedrock (~177 to 174 Ma) exposed at Sif Island in Pine Island Bay, which forms part of the topographic high between Pine Island and Thwaites glaciers (Fig. 1B) (*26*).

A Permo-Triassic age peak at ~275 to 230 Ma is also found in all the Holocene sediments analyzed (Fig. 2). Plutonic rocks bearing zircons of this age outcrop locally in the Kohler Range and at Mount Murphy (Fig. 1B) (*27*, *28*). Recycled zircons of this age are present in certain sedimentary Beacon Supergroup formations of the Transantarctic Mountains and are thought to be derived from a concealed former Pacific Gondwana margin magmatic arc in West Antarctica (*29*). The prominent ~275- to 230-Ma zircon age peak in all the Holocene samples lends support to this hypothesis, potentially pointing to a location beneath the Pine Island and Thwaites glacier catchments.

Further zircon U-Pb age populations in the Holocene samples include ~650- to 450-Ma, ~1100- to 850-Ma, and older zircons (Fig. 2). These zircons could originate from a concealed (minor) crystalline source but are more likely to be recycled from the metasedimentary units bearing Proterozoic–Early Phanerozoic detrital zircons that are present locally (Fig. 1). The population spanning ~1100 to 850 Ma is not discussed further because it has little value as a provenance tracer; such ages are ubiquitous in sedimentary deposits throughout West Antarctic and the Transantarctic Mountains, with no clear spatial trend in abundance or age. The small population of ~650 to 450 Ma ages in the mid-Cretaceous sediments underlying the Polarstern Sandstone indicate that ~650- to 450-Ma zircons were available at the land surface locally before the Cenozoic and, thus, at the time of Polarstern Sandstone deposition (Figs. 1B and 2E). This is supported by a 450- ± 17-Ma zircon U-Pb age found in Cretaceous sediments exposed in the Kohler Range (*n* = 11) (*30*).

### Provenance approach

The new Holocene detrital zircon U-Pb data help elucidate the characteristics of subglacial rock groups around the Amundsen Sea Embayment. We next revisit the provenance characteristics of the Polarstern Sandstone (*14*) alongside our new data and compare them to a compilation of published bedrock and detrital zircon U-Pb datasets from West Antarctica and the Transantarctic Mountains. This permits identification of likely source rocks for the Polarstern Sandstone. The spatial distribution of diagnostic bedrock sources is assessed, including the presence/absence of key age populations and the distribution of dates within these populations. When provenance indicators are nonunique and detritus could be sourced from multiple locations, we assess whether there is a requirement for long-distance transport or whether local transport can explain the data (*31*).

Because unconsolidated Quaternary deposits—such as glaciomarine sediments, subglacial tills, or terrestrial moraines—naturally integrate the surrounding geology, they partially account for uncertainties such as subglacial extent of rock types and their relative zircon fertility. This is evidenced by the broad agreement between moraine samples on land and offshore glaciomarine sediments in the Ross Sea (*32*). Unconsolidated deposits may also reveal the presence of rock types not currently exposed. These unconsolidated sediments are therefore preferred for comparison to the Polarstern Sandstone. However, in regions where these deposits are unavailable, like much of Marie Byrd Land, we opt for comparison to data from rocks.

### Source of the Cretaceous (~120 to 90 Ma) detrital zircon U-Pb ages and apatite U-Pb ages

**Fig. 4.**
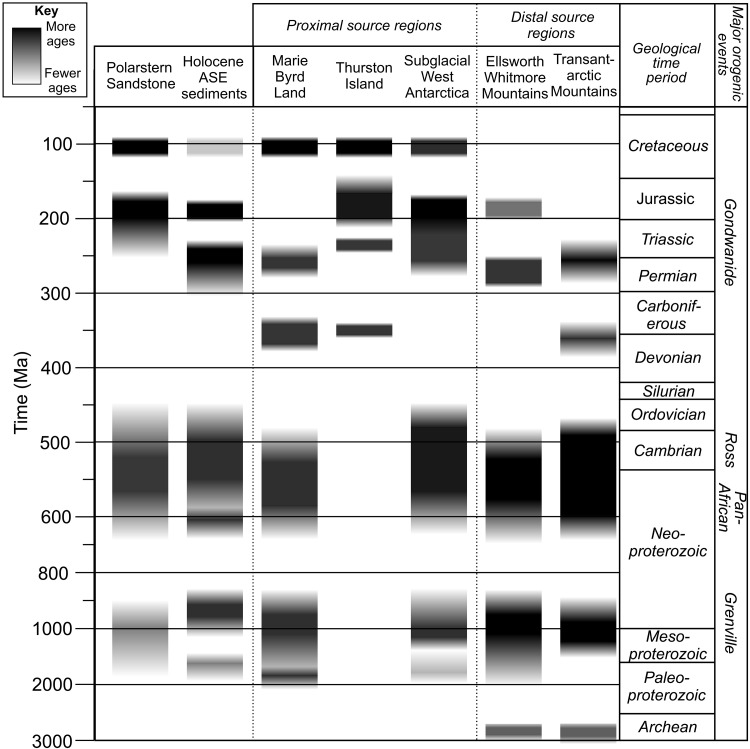
Relative age range and abundance of zircon U-Pb ages sourced from different regions of West Antarctica. These qualitative interpretations are based primarily on exposed geology (*95*) and knowledge of zircon fertility from unconsolidated sediments where these data are available. The “Subglacial West Antarctica” region does not contain any rock exposures. Entries for this therefore draw upon data from till beneath the Siple Coast ice streams (*34*) and debris from the base of the Byrd ice core in central West Antarctica (*10*), alongside Last Glacial Maximum to Holocene marine sediments deposited in the Ross Sea (*34*, *46*) and Holocene Amundsen Sea sediments (*21*, *43*, this study).

The first line of evidence for a local sediment source for the Polarstern Sandstone comes from the major input of detritus from Cretaceous bedrock (Figs. 1 and 2). Ages of ~120 to 90 Ma are abundant in zircon and apatite U-Pb data, as well as zircon U-Pb–dated rhyolitic pebbles (Fig. 2) (*14*). Notably, Cretaceous zircon and apatite U-Pb ages are entirely absent from rocks and moraines in the Transantarctic Mountains (*32*–*37*) and the Ellsworth-Whitmore Mountains block (Fig. 4) (*38*–*42*). Known Cretaceous magmatism in Antarctica was limited to the Antarctic Peninsula, Thurston Island, Marie Byrd Land, and the West Antarctic Rift System (*23*).

In the Polarstern Sandstone, similarities are identified between the Cretaceous rhyolite pebbles present and rhyolites exposed in the Jones Mountains (Fig. 1) (*14*). Furthermore, low-lying islands expose granitoids on the east side of Pine Island Bay with zircon U-Pb ages spanning ~127 to 94 Ma (*25*). Therefore, the ~120- to 90-Ma-old zircons could derive from the Thurston Island crustal block. Cretaceous crystalline bedrock also forms exposures in Marie Byrd Land to the west (Fig. 1), and these rocks likely extend beneath parts of the modern-day ice sheet. Cretaceous zircon U-Pb ages are found beneath Bindschadler and Kamb ice streams (*34*) and in the Byrd ice core basal debris (*10*). Detrital minerals from Amundsen Sea shelf sediments also yield Cretaceous ages (Fig. 2) (*21*, *43*), although rocks bearing Cretaceous zircons appear to be rare beneath eastern Thwaites Glacier (Fig. 2A). As Cretaceous rocks appear to be the largest contributor to the Polarstern Sandstone (*14*), they point to a dominant local source (Fig. 4).

### Source of zircon with Jurassic (~190 to 160 Ma) U-Pb ages

The second most prominent zircon U-Pb age peak from the Polarstern Sandstone dates to the Jurassic (~175 Ma; Fig. 2B) (*14*). These grains are most likely derived from Jurassic igneous bedrock exposed adjacent to Pine Island Bay (*25*) and between Thwaites and Pine Island glaciers (Fig. 1) (*26*). The wider extent of the Jurassic granitoids beneath the modern catchments of Thwaites and Pine Island glaciers is indicated by geophysical data (*44*), as well as Jurassic detrital biotite and hornblende ^40^Ar/^39^Ar ages (*21*) and zircon U-Pb ages in marine sediments from the Amundsen Sea Embayment (Figs. 1 and 2). The εHf values of Jurassic zircons in the Polarstern Sandstone span ~+5 to −10 (*14*), which closely match values from Sif Island, where a 177- to 174-Ma-old granite is exposed (*26*). Thus, the Jurassic grains in the Polarstern Sandstone are very likely to be locally sourced.

The maximum southerly extent of the bedrock source for the Jurassic zircons is difficult to constrain, but it probably extends into the West Antarctic interior and towards the Ross Sea. Granites of this age are exposed in the Whitmore Mountains far to the south (Fig. 1) (*45*). Triassic-Jurassic detrital zircons are also found in the basal debris of the Byrd ice core near the ice divide between the Ross Sea and Amundsen Sea sectors (Fig. 1A) (*10*), beneath some Siple Coast ice streams (*34*), and in Ross Sea sediments (*34*, *46*). However, these distant source regions are considered less likely than the Jurassic rocks present directly adjacent to the Amundsen Sea Embayment (*31*).

### Absence of a Permo-Triassic (~275 to 230 Ma) age peak

A notable difference between Holocene Amundsen Sea Embayment sediments and the Polarstern Sandstone is the presence of ~275- to 230-Ma zircon U-Pb ages in all Holocene sediments (Fig. 2). A large ~275- to 230-Ma age peak is also present in the Cretaceous sediments recovered from the same site (PS104_20-2 10R-1W; Fig. 2E). From these shared peaks, it seems very likely that rocks yielding ~275- to 230-Ma zircons would have been present at the land surface locally to the core site during the Eocene. However, the Polarstern Sandstone has a low yield of these zircon U-Pb dates: only 4% of grains (*14*).

**Fig. 5.**
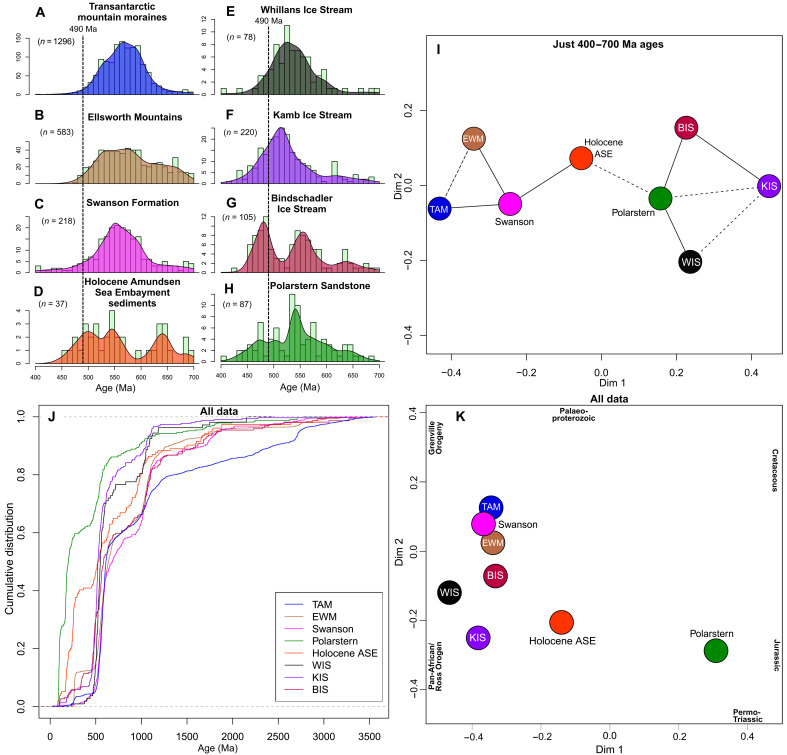
Comparisons between zircon U-Pb data for the Polarstern Sandstone, our new Amundsen Sea Embayment data, and regional sources known to bear Late Proterozoic to Ordovician ages. To examine the distribution of ages within the Late Proterozoic to Ordovician population, (**A**) to (**H**) display the data from 700 to 400 Ma only as kernel density estimates with a 12-Myr bandwidth overlain on 10-Myr bin histograms using IsoplotR (*92*). The vertical dashed lines plotted at 490 Ma highlight the relative abundance of grains younger than this age. Data compiled for (A) Transantarctic Mountains (TAM) moraines (*32*, *33*, *35*, *36*, *65*), (B) Ellsworth-Whitmore Mountains (EWM) sedimentary rocks (*37*–*40*), (C) the Swanson Formation and nearby metasediments (*101*, *102*), (D) Holocene sediments from the Amundsen Sea Embayment (ASE, this study), (E) subglacial sediments from Whillans Ice Stream (WIS) (*34*), (F) Kamb Ice Stream (KIS) (*34*), (G) Bindschadler Ice Stream (BIS) (*34*), and (H) the Polarstern Sandstone (*14*). (**I**) A multidimensional scaling (MDS) plot of the same 400- to 700-Ma data, where points plotted closer together can be interpreted as more similar and those further apart as less similar. Solid lines join nearest neighbors and dashed lines second nearest neighbors. (**J**) A cumulative age distribution plot for the full age range (0 to 3500 Ma), highlighting the sizeable population on Mesozoic ages in the Polarstern Sandstone. (**K**) An MDS plot of the full age range (0 to 3500 Ma). Bold text indicates the direction of influence of unimodal synthetic age populations with mean ages of 100 Ma (Cretaceous), 175 Ma (Jurassic), 250 Ma (Permo-Triassic), 550 Ma (Ross/Pan-African Orogeny), 1000 Ma (Grenville Orogeny), and 2050 Ma (Paleoproterozoic). These age populations were added to visualize which age peaks influence the locations of samples in the plot. Both MDS plots use the Kolmogorov-Smirnov dissimilarity measure, with dimensionless axes.

The most likely explanation for this is that the river catchment supplying detritus to the Polarstern Sandstone did not erode the source area of the ~275- to 230-Ma-old rocks. As the large catchment of a continental-scale river system would be expected to cover all the lithologies exposed, the lack of an ~250-Ma age peak in the Polarstern Sandstone (and the lack of an ~175 Ma age peak in the mid-Cretaceous mudstone) point to their deposition in more localized drainage basins. In addition, some sedimentary formations of the Transantarctic Mountains and Ellsworth-Whitmore Mountains contain ~270- to 240-Ma zircon U-Pb populations (Fig. 4 and 5J) (*28*, *39*, *47*, *48*). Although it is possible that localities bearing rocks or sediments with this zircon U-Pb age population may have coincidentally fallen outside the catchment, the absence of this population in the Polarstern Sandstone argues against long-distance sediment transport from these regions.

Extensive ice cover makes it impossible to accurately constrain the extent of potential ~275- to 230-Ma source rocks. However, hornblende and biotite mineral grains with these Permo-Triassic ages emerge from beneath Pine Island Glacier and Thwaites Glacier (Fig. 2) (*21*), and rocks of this age are exposed to the west in the Kohler Range and at Mount Murphy (Fig. 1B) (*27*, *28*). A Thurston Island crustal block source therefore seems most likely for the Polarstern Sandstone. Potentially, the switching from the ~275- to 230- to ~190- to 160-Ma age populations at Site PS104_20-2 from the Cretaceous to the Eocene may be linked to local fault-controlled subsidence and uplift altering sediment routing. The Thurston Island/Marie Byrd Land crustal block boundary (beneath Thwaites Glacier) may have been active in the Cretaceous, and Pine Island Rift (beneath Pine Island Glacier) saw a vertical offset exceeding 1 km at ~100 to 90 Ma (*26*). A source beneath the modern Pine Island and Thwaites glaciers may have provided ~275- to 230-Ma zircon grains during the ~92- to –83-Ma deposition of the Cretaceous sediments analyzed here. Continued tectonism and exhumation then led to a shift to routing from the Thurston Island crustal block by the Eocene, delivering ~190- to 160-Ma zircons to the Polarstern Sandstone depocenter.

### Neodymium and Strontium isotope compositions

The neodymium (Nd) and strontium (Sr) isotope compositions of the Polarstern Sandstone offer a further provenance characteristic to constrain potential source regions. Measurements from the Polarstern Sandstone give mean ε_Nd_ values of −3.8 and ^87^Sr/^86^Sr ratios of 0.718 (*n* = 2) (*15*). These Nd and Sr isotope compositions are most similar to marine surface sediments from Thurston Island and Thwaites Glacier (ε_Nd_ ≈ −4, ^87^Sr/^86^Sr ≈ 0.714) and differ compared to Pine Island Glacier–derived detritus (ε_Nd_ ≈ −9, ^87^Sr/^86^Sr ≈ 0.728) (*21*, *43*). Moraine samples integrating detritus from various Transantarctic Mountain rock types also give very different Nd and Sr isotope compositions (mean ε_Nd_ ≈ −13, ^87^Sr/^86^Sr ≈ 0.726) (*49*, *50*). The Nd and Sr isotope data therefore suggest that the Polarstern Sandstone consists primarily of material delivered by the more radiogenic Mesozoic rocks from either the Thurston Island or Thwaites Glacier regions, with the Pine Island Glacier catchment and distant provinces such as the Transantarctic Mountains very unlikely to be major sources.

### Source of zircon, rutile, and apatite grains with Late Proterozoic–Ordovician (~650 to 450 Ma) U-Pb dates

Detrital zircons, rutile, and apatite grains with Late Proterozoic–Ordovician (~650 to 450 Ma) U-Pb age peaks are present in the Polarstern Sandstone, with proportions of 20, 72, and 12% of dated grains for each respective mineral (Fig. 2D) (*14*). Locally exposed metasediments of Late Proterozoic-Ordovician age are potential source rocks. A paragneiss exposed near Mount Murphy and on Bear Peninsula has detrital zircon U-Pb age spectra peaking at ~500 Ma, and a paragneiss from Clark Island in the Amundsen Sea Embayment has a Rb-Sr isochron age of 446 ± 12 Ma (*27*). Clast counts in box core sediments from the eastern Amundsen Sea Embayment indicate the existence of a local source of low-grade metasedimentary rocks subglacially (*51*). Furthermore, metasediments of likely Late Proterozoic–Ordovician age have been inferred from geophysical data to be the probable subglacial basement between igneous intrusions in this region (*44*). Metasedimentary sources proximal to the Amundsen Sea Embayment could therefore be the source of the ~650- to 450-Ma detrital zircon, rutile, and apatite grains found in the Polarstern Sandstone.

A local subglacial source of Late Proterozoic–Ordovician grains is further supported by the notable population of ~650- to 450-Ma-old zircons in all measured Holocene sediments from the Amundsen Sea Embayment (~20 to 25%; Figs. 2 and 5J). These cannot be entirely recycled from older Eocene-Pleistocene sediments because they are also found, albeit with relatively low abundance, in the Cretaceous (~92 to 83 Ma) mudstone beneath the Polarstern Sandstone (Fig. 2E), implying that they were locally available during the Eocene.

To assess the source of the Late Proterozoic–Ordovician zircons and further examine the possibility of long-distance transport, we examined the distribution of dates from literature sources between 700 and 400 Ma, which spans the Pan-African (~650 to 550 Ma) and Ross (~550 to 450 Ma) orogenies (Fig. 5). Pan-African Orogeny zircon U-Pb ages spanning ~650 to 550 Ma can be diagnostic. These ages are more common in tills from the Transantarctic Mountains (34%) and rocks of the Swanson Formation (26%) and the Ellsworth-Whitmore Mountains (23%). They are rarer in tills underlying Siple Coast ice streams (16%) and entirely absent from the basal debris of the Byrd ice core (*n* = 16; Fig. 1A). In the Polarstern Sandstone, 650- to 550-Ma zircon U-Pb ages comprise only 8% of grains (Fig. 5) (*14*). A low abundance of ~650- to 50-Ma ages is therefore most consistent with subglacial source locations in West Antarctica (Figs. 4 and 5).

The proportion of zircons dating to the latest part of the Ross Orogeny, ~490 to 450 Ma, provides further constraint on source regions. The 490- to 450-Ma ages are very rare in moraines from the Transantarctic Mountains, rocks from the Ellsworth-Whitmore Mountains, and the Swanson Formation, where they comprise <2, 3, and 4% of zircons in the 700- to 400-Ma age range, respectively (Fig. 5). Rocks bearing zircons with U-Pb dates younger than 490 Ma are present in the southern Transantarctic Mountains, but such dates are rare relative to zircons older than 490 Ma and there are no well-dated plutons younger than ~475 Ma or metasedimentary rocks with such a young age population (*52*). Late Ross Orogeny ages are, however, present in tills from beneath Bindschadler and Kamb ice streams (comprising 33 and 20% of 700- to 400-Ma ages, respectively; Fig. 5), despite a lack of rock exposures bearing zircons of this age range (*34*). This 490- to 450-Ma age range is also present in the Byrd ice core debris, although the number of grains analyzed is small (three of eight 700 to 400 Ma ages) (*10*). In the Polarstern Sandstone, the substantial Late Ross Orogeny (490 to 450 Ma) age population (15% of 700- to 400-Ma grains) therefore hints toward a West Antarctic subglacial source region bearing these ages (Fig. 5H). This source area includes the upper portion of the modern Bindschadler/Kamb ice stream catchments and likely extends beneath the modern Thwaites Glacier catchment based on 490- to 450-Ma ages forming 11% of 700- to 400-Ma ages in Holocene Amundsen Sea Embayment sediments (Figs. 2, 5D).

The abundance of Late Ross Orogeny zircons (~490 to 450 Ma) and scarcity of Pan-African Orogeny zircons (~650 to 550 Ma) is most consistent with a West Antarctic subglacial source of the Late Proterozoic–Ordovician grains in the Polarstern Sandstone. At the time of writing, no published εHf values from zircons of this age are available from subglacial source rocks in West Antarctica to allow for comparison to εHf values in Polarstern Sandstone detrital zircons (*14*). The range of zircon εHf values in the Polarstern Sandstone is, however, broadly similar to that seen in Late Proterozoic–Ordovician zircons from the Ellsworth-Whitmore Mountains block (*28*, *38*) [we note that the field for this population is plotted ~90 Ma too old in reference (*14*), their Fig. 5A]. More negative εHf values of <~−10, as recorded in the Central Transantarctic Mountains (*52*), are also absent in the Polarstern Sandstone. This suggests that the inferred local and subglacial sources of Late Proterozoic–Ordovician zircons have a greater εHf affinity with the West Antarctic Ellsworth-Whitmore Mountains block.

Rutile grains from the Polarstern Sandstone also yield Late Proterozoic–Ordovician U-Pb ages, peaking at ~543 Ma (*14*). Given the ~600°C rutile U-Pb closure temperature, its propensity to be formed mainly in metamorphic and hydrothermally altered rocks, and its scarcity in nonultramafic igneous rocks, detrital rutile can be used to provide a record of medium- to high-grade metamorphic events in the sediment source region (*53*). The dominant ~543-Ma rutile U-Pb age peak in the Polarstern Sandstone therefore likely records either Late Pan-African or Early Ross Orogeny metamorphism (*14*). However, bedrock rutile U-Pb dating has not been widely applied in Antarctica, limiting its current provenance applications as potential source rocks and sediments have not been analyzed to compare to the Polarstern Sandstone data. This means that the fertility of rutile in regional rocks and the sensitivity of this provenance proxy to different West Antarctic metamorphic and magmatic events are uncertain. Furthermore, like zircon, rutile can survive multiple sedimentary cycles. These data therefore currently offer little constraint on sediment provenance in West Antarctica, although wider application of rutile U-Pb dating could yield useful results in the future.

In summary, the large geographical spread of Late Proterozoic–Ordovician (~650 to 450 Ma) source rocks (Fig. 1) hinders use of these mineral grains to identify the provenance of the Polarstern Sandstone (*14*). However, the distribution of ages within this population is most compatible with a source from local West Antarctic rocks (see also “Excluding the Transantarctic Mountains as a source area” section).

### Source of the detrital apatite grains with Triassic-Jurassic fission track ages

Seventeen of the 23 apatite grains from the Polarstern Sandstone dated using the AFT method gave ages of <140 Ma (*14*), which are very common throughout Marie Byrd Land and Thurston Island (*26*, *51*, *54*–*56*). Apatite U-Pb dates for most of these grains are ~100 Ma, consistent with a local West Antarctic source (*14*). However, six apatite grains (26%) from the Polarstern Sandstone have older Triassic-Jurassic (>150 Ma) AFT ages. While two of these six grains have U-Pb ages markedly younger than their AFT ages (98 and 144 Ma), suggesting an issue with the AFT ages, the four remaining AFT ages >150 Ma are somewhat unusual in West Antarctica, with potential value as a provenance tracer.

Grains with Triassic-Jurassic AFT dates could feasibly derive from the Heritage Range of the Ellsworth Mountains, where one AFT age (*57*) and several zircon (U-Th)/He ages (*58*) exceed 150 Ma. AFT ages of >150 Ma are also found more distally in Ross Orogen granites in the Thiel Mountains (Fig. 1A) (*59*) and morainal boulders in the central Transantarctic Mountains thought to be derived from the subglacial interior East Antarctica (*60*). However, bedrock sources bearing >150-Ma AFT ages may also be present subglacially close to the Polarstern Sandstone core site, as detrital apatite grains with such AFT ages are present in a similar proportion in nearby Holocene Amundsen Sea Embayment sediments (24% of 86 apatite grains from Site PS75_219-2) (*51*). Apatite is considered to primarily derive from first cycle detritus (*61*), but reworking of apatite from underlying Cretaceous sedimentary rocks cannot be excluded. We consider it most likely that rocks containing apatite with Triassic-Jurassic AFT ages are present locally/subglacially to provide detritus to Holocene sediments (*51*) and therefore the Polarstern Sandstone (*14*).

### Source of the hematite-cemented lithic arkose pebbles

Hematite-cemented lithic arkose pebbles found in the Polarstern Sandstone may also be a potentially distinctive provenance indicator (*14*). This unusual lithology is present in conglomerates of the Kukri Hills in the northern Transantarctic Mountains (Fig. 1A) (*14*). If this area was a unique source, the observation would require fluvial transport of a rock of moderate competency over more than 2000 km. However, other diagnostic Transantarctic Mountain lithologies—such as Ferrar Dolerite, Beacon Supergroup sediments, Shackleton Limestone, and Ross Orogeny granites—are notably absent from pebbles in the Polarstern Sandstone despite their availability in widespread and closer outcrops in the Transantarctic Mountains. Furthermore, preservation of lithic arkose pebbles during >2000 km of fluvial transport is unlikely. Transport of pebbles made of highly resistant lithologies, such as quartzite, over thousands of kilometers is possible (*62*), but a key characteristic of continental-scale river systems is that most coarse grains, such as gravel and pebbles, vanish within 10 to 40 km of entering a coastal plain, as is observed in the Himalayas (*63*). We consider the hematite-cemented lithic arkose pebbles most likely to be from a local source area that is currently covered by the WAIS. Detailed geochemical and geochronological analyses of these arkosic pebbles should be carried out in the future.

### Excluding the Transantarctic Mountains as a source area

Our new data and assessment of diagnostic provenance indicators show that rocks and sediments local to the Amundsen Sea Embayment provide a compelling match for the provenance characteristics of the Polarstern Sandstone, without the need for long-distance transport. Some provenance indicators are not unique and therefore cannot be used to trace the detritus in the Polarstern Sandstone to a specific geographic source region. In these instances, we consider the supply of this detritus from the nearest source region more likely than its delivery from a distal source area, bypassing the proximal source region (*31*). A longer transport distance, with a major proportion of Polarstern Sandstone detritus derived from a distal source in the Transantarctic Mountains, has, however, previously been argued (*14*). Below, we detail why this source region is unlikely.

First, the Jurassic (~175 Ma) zircon U-Pb ages in the Polarstern Sandstone have been suggested to derive from the Ferrar Group of the Transantarctic Mountains (*14*). This is extremely unlikely, as the Ferrar Group comprises basalts and dolerites that have a very low zircon fertility (*64*). This is reflected by the absence of zircons with ~180-Ma U-Pb ages from moraine samples in the Transantarctic Mountains, even when Ferrar Group rocks outcrop directly adjacent to these tills and are abundant in the pebble and cobble fraction (*36*). This lithologic bias in mineral fertility highlights the importance of comparing sediment provenance results from downstream regions to data from unconsolidated sediments close to upstream regions where such data are available (*33*, *35*, *36*, *53*, *65*). The latter data benefit from the integration of upstream source lithologies that accounts for factors such as the subglacial exposure of different rock groups and zircon fertility.

Second, the Late Proterozoic–Ordovician (~650 to 450 Ma) zircons in the Polarstern Sandstone have been attributed to primary erosion of Ross Orogen igneous rocks in the Transantarctic Mountains (*14*). However, the low proportion of Late Proterozoic–Ordovician (~650 to 450 Ma) zircon U-Pb ages in the Polarstern Sandstone argues against suggested transport from the Transantarctic Mountains (*14*). The proportion of this age population is very low in the Polarstern Sandstone (Fig. 2D) compared to Transantarctic Mountains sediments (Fig. 5J) (*29*, *35*, *36*) and western Ross Sea sediments, which have a dominant contribution from Transantarctic Mountains rocks (*66*–*68*). In an Eocene sedimentary erratic from the McMurdo region, Late Proterozoic–Ordovician zircons dominate the age spectra (*69*). The comparatively low abundance of Late Proterozoic–Ordovician detrital zircon ages in the Polarstern Sandstone (*14*) as well as the contrasting age distribution within this population (Fig. 5), therefore argues against anything more than an extremely minor contribution from the Transantarctic Mountains.

**Fig. 6.**
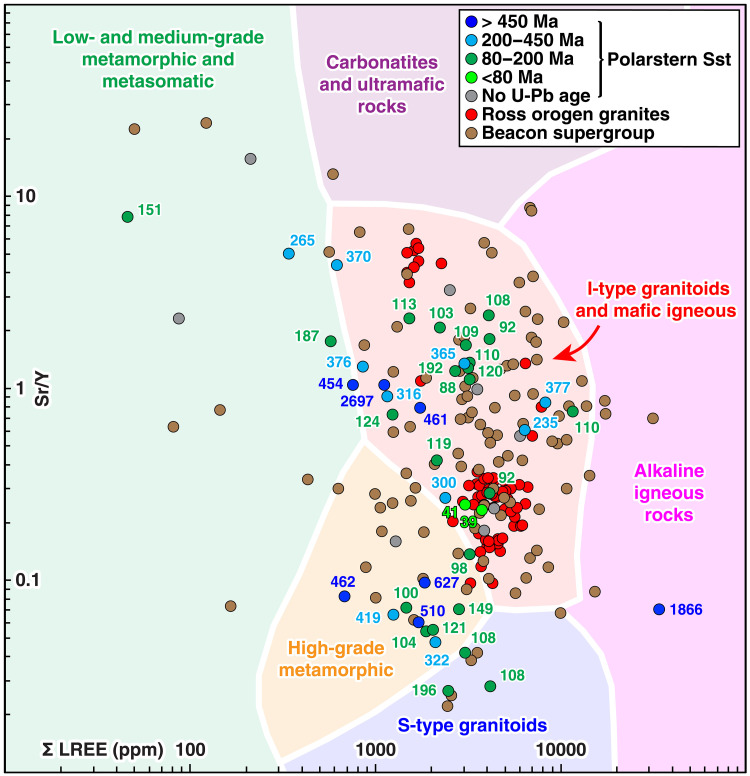
Polarstern Sandstone (detrital) and central Transantarctic Mountains (bedrock) apatite T/REE data plotted as sum of light REE (La, Ce, Pr, and Nd) versus Sr/Y concentrations [in parts per million (ppm)]. Data from the Polarstern Sandstone (Polarstern Sst) (*14*) mostly have corresponding same-grain apatite U-Pb ages, indicated with color-coded labels. Central Transantarctic Mountains bedrock apatite T/REE data (from solutions used for (U-Th)/He dating) (*72*) are from Cambrian Ross Orogen granitoids (~545 to 485 Ma) (*52*) and Permian to Jurassic sandstones of the Beacon Supergroup (>~200 Ma) (*29*). We do not include data for West Antarctic bedrock samples as none are available. The host rock lithology fields are decision boundaries based on a machine learning classification algorithm of a large apatite T/REE host-rock lithological database (*71*). Note that there is a layering error in the extent of the high-grade metamorphic and S-type granitoid fields in reference (*14*) (their Fig. 6A) that is corrected in this plot.

Furthermore, potential reworking of Late Proterozoic–Ordovician igneous zircons from a more proximal West Antarctic sedimentary source was ruled out by arguing that Paleozoic clastic deposits in West Antarctica (such as the Swanson Formation) have been affected by low-grade metamorphic overprinting (*70*), a process that can alter apatite grain trace element composition (*14*, *71*). Trace element patterns for most of the apatite grains analyzed from the Polarstern Sandstone suggest derivation from an igneous source (Fig. 6) (*14*). However, most of the Polarstern Sandstone detrital apatite grains with trace and rare earth element (T/REE) data that plot in the “I-type granitoid and mafic igneous” lithology field are either Cretaceous or Devonian-Carboniferous in age (Fig. 6). Cretaceous and Devonian-Carboniferous igneous rocks are far more common in West Antarctica compared to the Transantarctic Mountains (Fig. 1) (*23*, *27*). Overall, the detrital apatite T/REE and U-Pb data strongly favor a West Antarctic source for most of apatite grains in the Polarstern Sandstone.

To assess the source of the five older detrital apatite U-Pb ages typical of the Ross Orogeny (~650 to 450 Ma), the Polarstern Sandstone apatite T/REE data are compared to bedrock apatite T/REE data from Cambrian granitoids of the Granite Harbour Intrusives Group and Permian to Jurassic sandstones of the Beacon Supergroup in the central Transantarctic Mountains (Fig. 6) (*72*). The five apatite grains with 650- to 450-Ma U-Pb dates plot well way from the light REE versus Sr/Y T/REE bedrock granite values in the central Transantarctic Mountains, with three grains (and one additional apatite with a 419-Ma U-Pb age) plotting within the “high-grade metamorphic” lithology field (Fig. 6). Furthermore, three of the five older (~650 to 450 Ma) apatite grains have ages between 462 and 454 Ma, much younger than the main pulse of Ross Orogen magmatism (and earlier metamorphism) in the Transantarctic Mountains (~545 to 485 Ma) (*52*). No medium- to high-grade metamorphic rocks younger than Late Ross Orogen magmatism have been described from the Transantarctic Mountains. A far more likely source for these metamorphic apatite grains is therefore a high-grade metamorphic orthogneiss with a Rb-Sr isochron age of 446 ± 12 Ma exposed on Clark Island, very close to site PS104_20-2, or subglacial equivalents (*27*).

Zircon εHf values and model ages from the Polarstern Sandstone were also concluded to have an affinity with zircons from the Transantarctic Mountains (*14*). However, as described above, most—or all—of the zircons from the Polarstern Sandstone have U-Pb ages more compatible with local West Antarctic rocks. The similarity between the Hf isotope model ages of the Transantarctic Mountains and Polarstern Sandstone suggested in the multidimensional scaling plot of (*14*) is therefore most likely a coincidence because a Hf isotope model age is recording the time when a melt was originally separated from its mantle source, which may not vary much geographically (*73*).

### Viability of a West Antarctic transcontinental river system

The low-relief model hypothesizes that paleo-river flows over a >1500 -km course between the Transantarctic Mountains and the Amundsen Sea, which requires low topographic relief (Fig. 3F). Beyond the provenance arguments, there are three key arguments for why this is unlikely from a geomorphological perspective.

First, in the simplest case, rivers tend to take the steepest, most direct route to base level at the coast. In the case of drainage that originated from the front of the Transantarctic Mountains (or a lower-relief precursor before onset of rapid exhumation at ~35 Ma) (*72*), any Eocene rivers would be expected to have flowed straight to the Ross Sea coast. At (and before) the Eocene-Oligocene boundary, the Ross Sea coastline was likely situated parallel and proximal to the location of the modern-day northern and central Transantarctic Mountains front (e.g., Fig. 3), as is the case today (Fig. 1). In the low-relief model, the upper tributaries of the proposed river network directly flow into the Ross Sea embayment and then are diverted south and across the interior of West Antarctica (*14*). Such a diversion would have required a strong paleotopographic control, such as a barrier of high topography stretching between the modern Ford Ranges and the Kukri Hills (Fig. 1A), to steer the rivers inland and away from the nearest coast. This interpretation, however, contradicts offshore sediment stratigraphy and backstripping analysis from the Ross Sea continental shelf, which indicates low-lying sedimentary depocenters and intervening structural highs situated close to sea level in the Late Eocene (*9*, *74*).

Thermochronological and geomorphological evidence from the Ford Ranges provides further evidence for tectonic quiescence, with a lack of notable relief and protracted erosion forming fluvial planation surfaces during the Eocene (*55*, *75*). Post–34-Ma sediment volumes derived from seismic profiles from both the Ross and Amundsen Seas (*76*, *77*) indicate that there is an insufficient volume of offshore detrital material to support the existence of an actively eroding area of continuous high topography in the Ross Sea region during or after the Eocene (*4*, *6*). This is the case even allowing for the large uncertainties associated with sediment volume, density, porosity, and biogenic versus terrigenous fraction (*6*). These lines of evidence indicate that no obvious topographic impediment existed to deflect rivers draining from the vicinity of the modern-day Transantarctic Mountain front toward the West Antarctic interior rather than to the proximal Ross Sea coast (Fig. 3).

Second, the West Antarctic Rift System had been active since the Late Cretaceous and Paleocene (*5*), forming structurally controlled topographic basins (grabens) (*11*) that were at least partly below local sea level as early as 47 to 45 Ma (*8*). Such basins are not fully resolved in preglacial paleotopographic reconstructions but are mapped as basement topography within the modern subsided crust (*11*). Although West Antarctic Rift System extension continued after deposition of the Polarstern Sandstone (at least in the western Ross Sea) (*78*, *79*), it is likely that structural features associated with rifting were already present in the Eocene. A transcontinental river system (as in the low-relief model) would have to have traversed perpendicular to the structural trend of these basins and basement highs; although possible, this is unlikely.

Third, a substantial topographic barrier (i.e., drainage divide) is present across central West Antarctica in all paleotopographic reconstructions for the Eocene-Oligocene transition (Fig. 3) and in the modern-day topography when isostatically adjusted for ice sheet removal. The Transantarctic Mountains also likely had much lower relief and, possibly lower elevation before the Oligocene, given that onset of rapid denudation in the central Transantarctic Mountains occurred at or immediately before ~34 Ma (*72*) as the Antarctic Ice Sheet grew. Thus, if these reconstructions are correct, the transportation pathway of siliciclastic material from the proto-Transantarctic Mountains front to the Polarstern Sandstone core site would have had to cross a central West Antarctic drainage divide flowing uphill.

We therefore suggest that during the Middle-Late Eocene, it is most likely that short, steep rivers drained the proto-Transantarctic Mountains directly to base (sea) level in the Ross Sea (*6*). Rivers draining to the Amundsen Sea would most likely have headwaters originating in West Antarctica. The low-relief model, with a West Antarctic transcontinental river system, cannot be fully excluded solely on the basis of these geomorphological arguments, but it would have specific, atypical paleotopographic requirements.

Geomorphological arguments alone cannot exclude the low-relief model and transcontinental river, so we rely on our simulated hydrological networks on possible West Antarctic topographies (Fig. 3) together with the available provenance data. Our new zircon U-Pb data, together with reevaluation of published geochronological, thermochronological, and geochemical data, indicate that supply of detritus from local rock types can explain all the provenance features of the Polarstern Sandstone (Figs. 4 and 5 and Table 1). All the Polarstern Sandstone’s components are available in rocks within approximately 800 km of the drill site and possibly much closer. A preferred source region is the Thurston Island block to the east, possibly extending into the West Antarctic interior. A very minor detrital contribution from more distal sources such as the Ellsworth-Whitmore Mountains or southernmost Transantarctic Mountains—possibly reworked through pre-Eocene sedimentary rocks—cannot be entirely ruled out but is not required to explain the provenance signature of the Polarstern Sandstone. In summary, local source areas can explain all the features of the sediment provenance data, a transcontinental river system is unlikely from a geomorphological perspective, and our hydrological simulations upon modeled Late Paleogene topography do not form a transcontinental river (Fig. 3). The most parsimonious solution is therefore that a river system crossing West Antarctica did not exist during the Middle-Late Eocene.

**Table 1.** Main provenance features of the Polarstern Sandstone. The previous provenance data interpretation (*14*) and our preferred interpretation, alongside a qualitative evaluation of the strength of each data type for source region identification.

Polarstern Sandstone provenance feature (*14*, *15*)	Reference (*14*) interpretation	Our interpretation	Strength of constraint on provenance
Common Cretaceous (~120 to 90 Ma) zircon U-Pb ages, apatite U-Pb ages, and rhyolitic cobbles.	Thurston Island and Marie Byrd Land crustal blocks (local).	Thurston Island and Marie Byrd Land crustal blocks (local).	Strong
Common Jurassic (~190 to 160 Ma) zircon U-Pb ages.	Ferrar Large Igneous Province (Transantarctic Mountains; distal)	Jurassic rocks exposed adjacent to the Amundsen Sea Embayment (local)	Strong
Near absence of Permo-Triassic (~250 Ma) zircon U-Pb ages.	N/A	Smaller river catchment, possibly confined to the Thurston Island crustal block (local).	Moderate
Mean ε_Nd_ values of −3.8 and ^87^Sr/^86^Sr ratios of 0.718.	N/A	Dominant contribution from West Antarctic Mesozoic rocks (local)	Strong
Late Proterozoic–Ordovician (~650 to 450 Ma) zircon, rutile, and apatite U-Pb age population.	Transantarctic Mountains granites (distal).	West Antarctic subglacial rocks/(meta)sediments (local, e.g. Clark Island).	Moderate (rutile U-Pb weak)
AFT ages spanning 213 to 24 Ma, including six Triassic-Jurassic (>150 Ma) ages.	Transantarctic Mountains (distal).	Local subglacial rocks; possibly moderately distant Ellsworth Mountains.	Moderate to weak
Unusual hematite-cemented lithic arkose pebbles.	Kukri Hills, northern Transantarctic Mountains (distal).	Unexposed/eroded subglacial rocks (local).	Weak
Apatite T/REE data	Igneous sources in the Transantarctic Mountains (distal).	Local Mesozoic igneous rocks and Late Proterozoic-Ordovician metamorphic rocks	Moderate

### Middle-Late Eocene West Antarctic topography

The existence of Middle-Late Eocene deltaic sediments fed by a river system nevertheless offer a useful constraint on paleotopography; Site PS104_20-2 must have been at or near sea level at ~44–34 Ma, with a region inland that was above sea level and supported a river network (*14*). Our modeled river networks on reconstructed paleotopographies (Fig. 3) help link the previously unidentified local geological source regions and the topographic features this river network would require.

Before interpreting these modeled drainage networks, it is important to consider that the Polarstern Sandstone could date to as early as ~44 Ma (*14*). Support for the Polarstern Sandstone recording sedimentation earlier in the 44- to 34-Ma interval is found in the record from the nearby core PS104_21-3 (location in Fig. 1), which contains evidence for shallow marine conditions at ~33.7 Ma (*3*). Site PS104_21-3 is located 60 km further offshore from site PS104_20-2, and the Amundsen Sea Embayment has a seaward dipping shelf stratigraphy (*15*). It is therefore unlikely that sediments at the two sites would have a similar age (~34 Ma). Although contrasting with the single 25.2 ± 6.9 Ma AFT age and 39.8 ± 2.1 Ma weighted mean of the two young apatite U-Pb ages (*14*), an age approaching 44 Ma for the Polarstern Sandstone may be most likely. This would imply an up to 10-Myr time difference between its deposition and the ~34-Ma topographic reconstructions (*4*, *6*). We judge that examining river networks on these paleotopographies is useful because the modeled topographies incorporate the best geologic evidence available at present from West Antarctica. They offer a direct means to assess the broad topographic features that influenced sediment provenance, transport, and depositional setting of the Polarstern Sandstone.

All the modeled river networks draining to the Amundsen Sea are confined to West Antarctica (Fig. 3). This is consistent with our provenance interpretations, which support the high-relief model with rivers draining into the Amundsen Sea having catchments confined to West Antarctica during the deposition of the Middle-Late Eocene Polarstern Sandstone. The provenance data do not require the transcontinental river of the low-relief model, nor the unlikely topographic steering of rivers from the Transantarctic Mountains inland, away from the Ross Sea (*14*).

The preglacial topographies of reference (*4*) show rivers draining part of the Thurston Island block and the West Antarctic interior beneath the modern Thwaites Glacier catchment (Fig. 3, D and E). These catchments are broadly compatible with the provenance and depositional setting constraints of the Polarstern Sandstone, with an elevated area immediately inland of the Amundsen Sea and a coastline near to the core site. However, the absence of an ~250-Ma zircon U-Pb age population argue against a dominant input from the modern Thwaites Glacier region, and these topographies (*4*) do not meet the requirement for a marine setting in parts of central West Antarctica during the mid-Eocene (Fig. 3) (*8*).

In the ~34-Ma median and maximum reconstructions of reference (*6*), the PS104_20-2 core site lies tens of kilometers inland of the coast on a strip of higher ground trending approximately east to west (Fig. 3, B and C). A shallow marine basin (100 to 200 m below modern sea level) is located inland (south) of the core site, with a deeper, more extensive marine basin (up to 300 m below modern sea level) in the location of the modern Pine Island Glacier catchment and draining toward the Weddell Sea. The modeled river catchments draining toward the Amundsen Sea cover parts of the Thurston Island block to the east and Thwaites Glacier region to the west (Fig. 3, B and C) and could therefore match the Polarstern Sandstone provenance signature. Although the core site is located slightly inland in these paleotopographies, it lies broadly within the coastal region where fluvio-deltaic sedimentation could occur.

There is an apparent discrepancy between the presence of the shallow marine basin to the south and the Polarstern Sandstone requirement for subaerial topography inland of the core site. Here, the ≤10-Myr time gap between Polarstern Sandstone deposition and the ~34-Ma topographic reconstructions may come into play. Over ~10 Myr, regional elevations would likely have been lowered by ~100 to 200 m with respect to sea level, primarily by thermal subsidence (*5*) but augmented by fluvial erosion (*80*). The ~34-Ma reconstructed elevations (*6*) therefore represent lower bounds for the time of Polarstern Sandstone deposition. The shallow marine basin south of the core site at ~34 Ma probably lay at or above sea level at ~44 Ma, supporting a river system flowing toward the inferred coastal deltaic depositional setting.

Further southeast, the deeper marine basin covering much of the location of the modern Pine Island Glacier catchment in the median and maximum reconstructions of reference (*6*) is unlikely to have been above sea level at ~44 Ma. This bedrock is submerged and unable to undergo erosion by any paleo-river network. Modern Pine Island Glacier substrate displays unradiogenic ε_Nd_ values (~−9) and a relatively high abundance of ^40^Ar/^39^Ar and U-Pb mineral ages spanning ~300 to 230 Ma (Fig. 2B) (*21*). These signatures are not observed in the Polarstern Sandstone (*14*, *15*). A marine basin over part of the Pine Island Glacier catchment in the Late Eocene, as in the reference (*6*) median and maximum 34-Ma topographies, may explain the lack of Pine Island Glacier–like detrital contribution to the Polarstern Sandstone.

The minimum paleotopography of reference (*6*) was configured to allow for the possibility that the Transantarctic Mountains were markedly lower at ~34 Ma. While their relief was very likely low, this reconstruction may not be correct as the mountain range could have had a high mean elevation (*72*). This reconstruction shows a deeper marine basin extending over the PS104_20-2 core site (Fig. 3A). This model does not, therefore, satisfy the coastal deltaic setting criteria implied by the Polarstern Sandstone record (*14*) and is not a feasible topography for the time of the Polarstern Sandstone deposition.

Our findings help elucidate the evolution of West Antarctic topography through the Middle-Late Eocene (up to ~44 Ma). We suggest that the Polarstern Sandstone was deposited by a river system draining a subaerial region around the Amundsen Sea, possibly confined to the Thurston Island crustal block and likely with a slightly higher topography than the reference (*6*) reconstructions at ~34 Ma due to the <10-Myr time gap. By ~33.7 Ma, the local area had transitioned to a shallow marine setting (*3*).

In summary, environmental conditions in the Amundsen Sea were temperate in the Middle-Late Eocene, with a locally subaerial West Antarctic topography capable of hosting local river systems flowing into a deltaic/coastal environment that deposited the Polarstern Sandstone (*14*). Our new zircon U-Pb data from Holocene and Cretaceous sediments in the Amundsen Sea Embayment “sample” the proximal bedrock. These data help test whether the higher-relief model drained by short rivers (*4*, *6*), or a low-relief model with a long (>1500 km) river system (*14*), is most valid for West Antarctica in the Middle-Late Eocene. Our detailed provenance analyses demonstrate that the characteristics of the Polarstern Sandstone can be fully explained by a short, local river system fed from nearby sources adjacent to the Amundsen Sea Embayment, with probable contributions from rocks currently beneath the WAIS. Hydrological modeling shows that an unusually long, flat river profile crossing perpendicular to the structural trends of an active rift system is unlikely. A river network with a drainage basin extending for no more than 800 km inland, and possibly considerably shorter, would conform with preglacial paleotopographic reconstructions showing a fluvial drainage divide situated in central West Antarctica and topography broadly sloping down toward the Ross and Amundsen seas. This scenario would be fully consistent with the constraints provided by the depositional setting and the provenance data from the Polarstern Sandstone. This topographic constraint offers fresh insight into the role of topography during climatic cooling and glaciation in West Antarctica, with implications for early ice-sheet reconstructions.

## MATERIALS AND METHODS

### Sample selection

To refine sediment provenance interpretation of the zircon U-Pb, zircon εHf, apatite U-Pb, and AFT data from the Polarstern Sandstone (*14*), it is useful to characterize the detritus eroded from bedrock sources proximal to the Amundsen Sea Embayment. We therefore measured the zircon U-Pb age composition of sediments in the Amundsen Sea Embayment close to the Polarstern Sandstone core site. No published detrital zircon U-Pb data for Holocene sediments in the Amundsen Sea Embayment previously existed.

Core NBP99-02_47PC is 75 cm long and was collected in 664-m water depth during R/V *Nathaniel B. Palmer* Cruise 99-02 in early 1999 (*81*). The core is described as silt-rich mud, containing fine to coarse sand and common (<25%), angular to subrounded, fine to very coarse pebbles (*81*). Zircons from three samples taken at depths of 14 to 18, 48 to 54, and 65 to 70 cm below sea floor were combined because of the low zircon yield. Although the pebble content increases toward the base of the core, the consistent glaciomarine lithology suggests that the sampled intervals were deposited during the mid-Late Holocene, postdating the last glaciation. They record a time of stable grounding-line position and sediment transport patterns since the Early Holocene (*82*). Detrital zircons from the three samples can therefore be safely treated as one.

The Thwaites Glacier grounding zone sample was recovered via a hot water–drilled access hole through the 587-m-thick Thwaites Eastern Ice Shelf (*83*). Sample recovery used a 1-m-long percussion gravity corer. The drill site is located on the 2011 “grounding line” (*84*), approximately 1.5 to 2.0 km downstream of the present-day grounding zone. Core barrel scrapings composed of glaciomarine sediment were used to ensure a large volume of material for provenance analyses. The sample is therefore an amalgamation of the uppermost ~1 m of glaciomarine sediment deposited during grounding line retreat since 2011. We assume that the Thwaites grounding zone sample does not show provenance change with depth and faithfully represents the “modern day” signature of Thwaites Glacier.

From box core NBP20-02_BC27, sediment was taken from a depth of 5 to 15 cm below sea floor. The sediment consists of silty clay. The sample is proximal and lithologically similar to the dated PIG-C core (*85*), so likely was deposited since Pine Island Glacier ungrounded from this location in the mid-Late twentieth century. It represents the modern day signature of Pine Island Glacier.

Last, we measured a sample taken from the Cretaceous mudstone recovered at Site PS104_20-2 from core 10R-1 W 111 to 126 cm. The sample lies stratigraphically below the Polarstern Sandstone (*15*). Zircon age populations in the mudstone present must have been available at the land surface and able to undergo transport and deposition before deposition of the Polarstern Sandstone. If Holocene sediments contain detrital zircons recycled from the Polarstern Sandstone during Cenozoic glaciation, this sample would help detect them.

### Analytical methods

The PS104_20-2 10R-1W 111- to 126-cm and NBP20-02 BC27 5- to 15-cm samples were sieved to 15 and 38 μm, respectively, to preserve the finer material for potential future applications. Because of the large sample volume, fines from the Thwaites Glacier grounding zone sample were suspended in water and decanted. Although our samples were therefore sieved to different minimum sizes compared to each other and literature data, this should not affect our zircon U-Pb age results because the minimum grain size analyzed is constrained by the laser spot diameter rather than the sieve size.

Zircons for the coarse fraction of the three samples were then concentrated using standard density separation and magnetic separation techniques at the London Geochronology Centre at University College London, and the entire separates mounted on slides in epoxy resin. We note that the Thwaites Glacier grounding zone sample was notably zircon-poor, with some ~190-Ma zircons proving slightly magnetic (i.e., in the 1- to 1.5-A magnetic separate). Zircon U-Pb dating for these three samples was performed at the London Geochronology Centre using an NWR193FX 193-nm laser with a 25-μm spot diameter coupled to an Agilent 7900 inductively coupled plasma mass spectrometer (ICP-MS). NIST612 glass was used to estimate U and Th concentrations (*86*). Plešovice zircon was used as a primary standard (*87*), with GJ1 (*88*) and 91500 (*89*) zircons used as secondary standards. Data reduction was performed using GLITTER 4.5 (*90*).

The site NBP99-02_47PC sample was sieved to 63 μm, and zircons from the coarse fraction were picked after being concentrated using standard density separation and magnetic separation techniques by ZirChron LLC, Tucson, AZ, USA. Zircon U-Pb dating was performed at the Arizona LaserChron Center (www.laserchron.org) following the methods of reference (*91*). A Photon Machines Analyte G2 excimer laser equipped with a HelEx ablation cell was used with a spot diameter of 15 μm, coupled to an Element2 HR-ICP-MS. Sri Lanka and FC-1 zircon crystals were used as the primary zircon standard reference materials and R33 zircon crystals were used as a secondary standard. Data reduction was performed in MATLAB using the Arizona LaserChron Center AgeCalcML software.

Data were processed and plots generated using IsoplotR (*92*). Discordant grains were rejected on the basis of the log ratio distance to the concordia composition, using a concordia distance filter of −2.3 to +9.4 which is approximately equivalent to a −5 to 20% relative age cutoff (*93*).

Because of these different data processing choices, the zircon U-Pb data from reference (*14*) displayed in plots here were reprocessed to ensure that there were no biases resulting from these different preferences. This produced negligible changes to the age populations present, although more grains were classified as discordant. Furthermore, we were unable to reproduce the peak ages previously reported (*14*). Our modal age estimates were based on visual inspection of the kernel density estimates, whereas the reported values (*14*) appear to correspond to the means of the subpopulations. For skewed distributions, such as the ~190- to 160-Ma population in the Polarstern Sandstone zircon U-Pb data, the mean and the mode can differ substantially. Our age peaks are rounded to the nearest 5 Myr to reflect that our estimates are influenced by the degree of smoothing applied to the kernel density estimate and the fact that it is difficult to estimate the peaks with precision, particularly for small samples.

### Hydrological modeling

We calculated the regional drainage networks for five paleotopographic reconstructions of West Antarctica (*4*, *6*) assuming that surface water is routed down the path of steepest descent within the landscape. All topographies are relative to present-day sea level. This calculation was performed using the TopoToolbox 2 MATLAB package (*94*). Before the hydrological routing calculation, internal sinks within the topographies were filled to remove enclosed topographic lows (basins) that would otherwise cause discontinuities in the calculated flow network. We chose an arbitrary threshold of 200 upstream cells to define an established hydrological pathway (equivalent to an area of 5 × 10^3^ km^2^ for the native grid resolution), which we found to be appropriate for capturing regional-scale drainage patterns. Although fluvial systems cannot flow below base level, we calculated drainage pathways as far out as the continental shelf edge, allowing us to examine the catchments in regions whose elevation may have been at or above sea level within the uncertainty of the topographic reconstructions and Eocene sea level.

To generate a heatmap of regions of the reconstructed paleotopographies that are upstream of the PS104_20-2 core site, we identified each grid cell situated within a drainage basin that has an outlet within a 100-km box centered on the core site (thereby allowing for a degree of uncertainty in the local topographic configuration that might cause pathways to pass close to, but not directly through, the single grid cell corresponding to PS104_20-2). The heatmap was computed by summing the number of grid cells meeting this criterion across the five modeled hydrological networks.
