# Supplementary material for: Reconstructing Eocene Antarctic river drainage from provenance analysis of Amundsen Sea embayment sediments
Source: Sci Adv. 2025 Dec 10;11(50):eaea2373. doi: 10.1126/sciadv.aea2373 (PMC12693975; doi:10.1126/sciadv.aea2373)
Supplement: Supplementary file 1 — Legends for data S1 and S2 [file sciadv.aea2373_sm.pdf]

Science Advances
AAAS

Supplementary Materials for

Reconstructing Eocene Antarctic river drainage from provenance analysis of Amundsen Sea embayment sediments

James W. Marschalek *et al.*

Corresponding author: James W. Marschalek, j.marschalek18@imperial.ac.uk

Sci. Adv. **11**, eaea2373 (2025)
DOI: 10.1126/sciadv.aea2373

The PDF file includes:

Legends for data S1 and S2

Other Supplementary Material for this manuscript includes the following:

Data S1 and S2

Data S1. (separate file): Zircon U-Pb data from Amundsen Sea Embayment samples.

Zircon U-Pb data from three Holocene sediment samples strategically located around the embayment to help characterise the detritus currently being eroded and deposited into the Amundsen Sea. Samples comprise of locations proximal to Pine Island Glacier and Thwaites Glacier, with a further sample to the north of Thwaites Glacier. A fourth sample consists of Cretaceous mudstone from a drill core from site PS104_20-2 in the Amundsen Sea Embayment.

Data S2. (separate file): Compiled detrital zircon U-Pb dates from potential source areas around West Antarctica.

Zircon U-Pb dates include the sedimentary rocks of the Swanson Formation and Ellsworth-Whitmore Mountains, as well as all known published West Antarctic subglacial till data. Data from moraines in the Transantarctic Mountains are also included.
